# Supplementary material for: An Insight into Goat Cheese: The Tales of Artisanal and Industrial Gidotyri Microbiota
Source: Microorganisms. 2023 Jan 3;11(1):123. doi: 10.3390/microorganisms11010123 (PMC9863150; doi:10.3390/microorganisms11010123)
Supplement: Supplementary file 1 [file microorganisms-11-00123-s001.zip › microorganisms-2122409-supplementary.pdf]

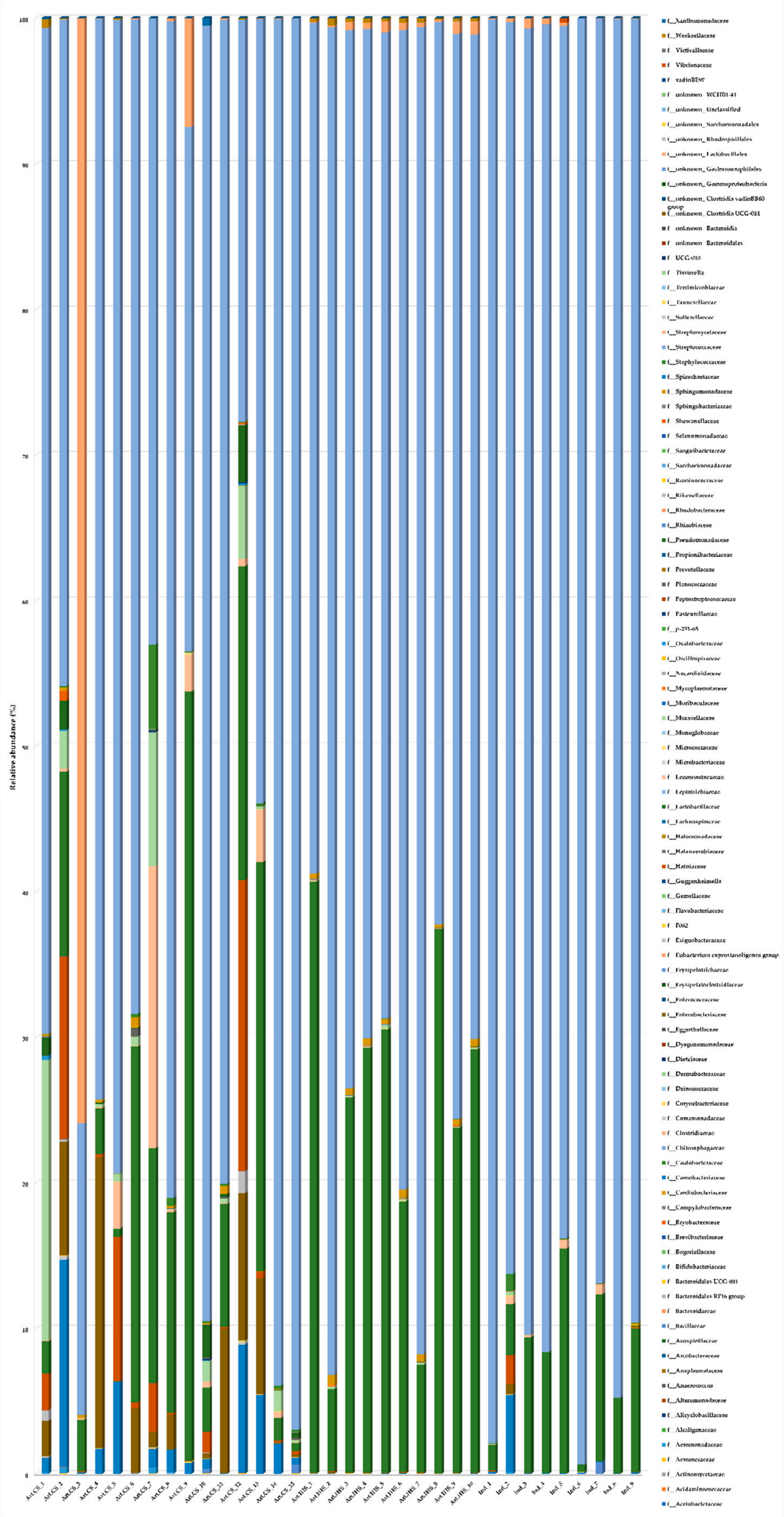

Figure S1. Stacked barplots of the relative abundance of the families identified in the Artisanal_CS (Art. CS), Artisanal_IHS (Art. IHS) and Industrial (Ind.) gidotyri cheeses.

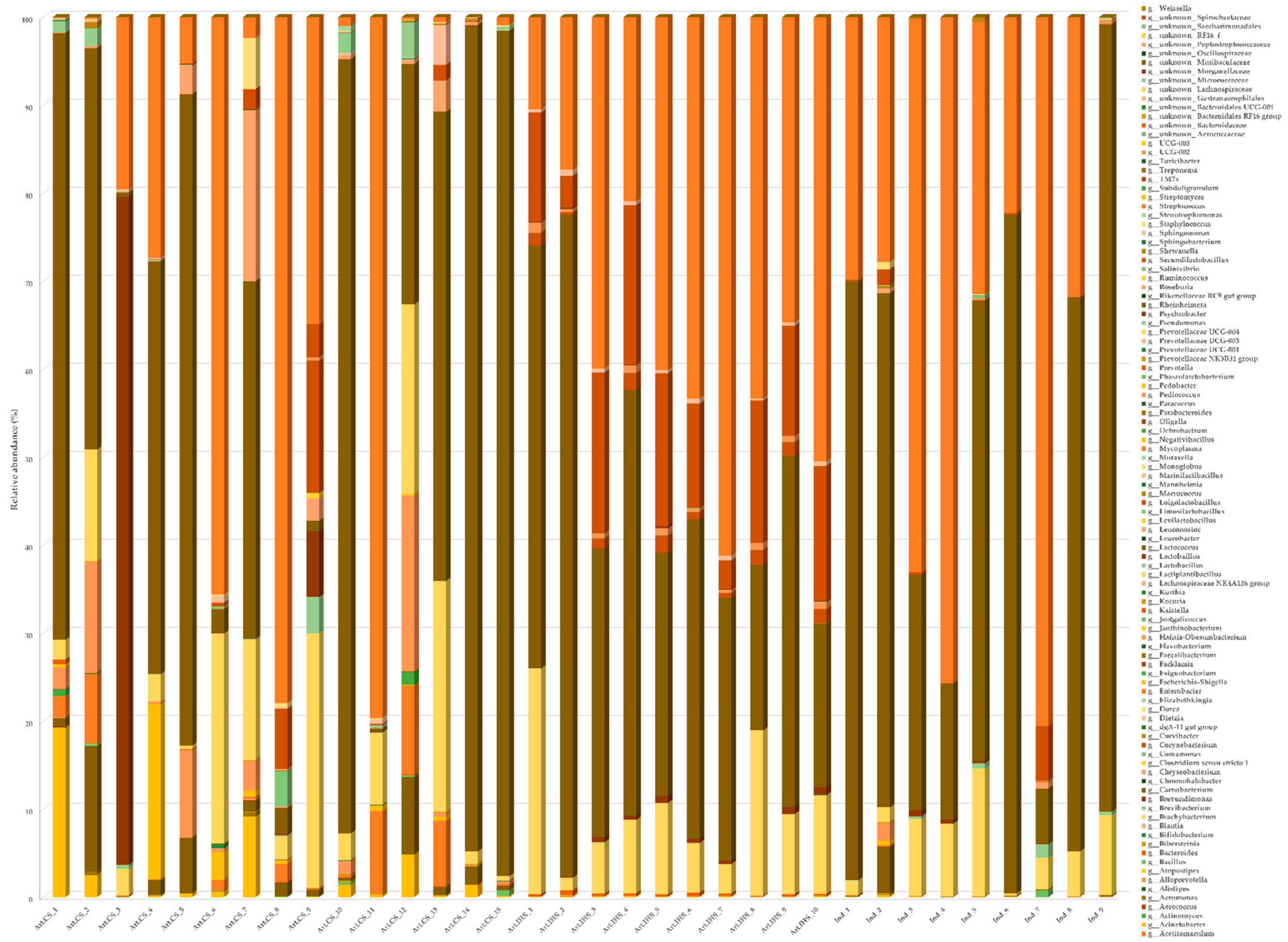

Figure S2. Stacked barplots of the relative abundance of the genera identified in the Artisanal_CS (Art. CS), Artisanal_IHS (Art. IHS) and Industrial (Ind.) gidotyri cheeses.
